# Guillain Barre Syndrome as a Complication of COVID-19: A Systematic Review

**DOI:** 10.1017/cjn.2021.102

**Published:** 2021-05-05

**Authors:** Mohammad Aladawi, Mohamed Elfil, Baha Abu-Esheh, Deaa Abu Jazar, Ahmad Armouti, Ahmed Bayoumi, Ezequiel Piccione

**Affiliations:** Department of Neurological Sciences, University of Nebraska Medical Center, Omaha, Nebraska, USA; Department of Neurology, Mercy Hospital, Oklahoma City, Oklahoma, USA; Department of Neurology, University of Texas Medical Branch – Galveston, Galveston, Texas, USA; Department of Neurology, University of South Florida Morsani College of Medicine, Tampa, Florida, USA; Department of Neurology, Yale University, New Haven, Connecticut, USA

**Keywords:** Guillain Barre syndrome, GBS, Miller Fisher syndrome, MFS, SARS-CoV2, COVID-19

## Abstract

**Background:**

In January 2020, the first case of Guillain Barre syndrome (GBS) due to COVID-19 was documented in China. GBS is known to be postinfectious following several types of infections. Although causality can only be proven through large epidemiological studies, we intended to study this association by a thorough review of the literature.

**Methods:**

We searched PubMed, EMBASE, and Google scholar and included all papers with English or Spanish full text and original data of patients with GBS and recent COVID infection. Variables of interest were demographics, diagnostic investigations, and the latency between arboviral and neurological symptoms. Further variables were pooled to identify GBS clinical and electrophysiological variants, used treatments, and outcomes. The certainty of GBS diagnosis was verified using Brighton criteria.

**Results:**

We identified a total of 109 GBS cases. Ninety-nine cases had confirmed COVID-19 infection with an average age of 56.07 years. The average latency period between the arboviral symptoms and neurologic manifestations for confirmed COVID-19 cases was 12.2 d. The predominant GBS clinical and electromyography variants were the classical sensorimotor GBS and acute demyelinating polyneuropathy respectively. Forty cases required intensive care, 33 cases required mechanical ventilation, and 6 cases were complicated by death.

**Conclusions:**

Studies on COVID-19-related GBS commonly reported sensorimotor demyelinating GBS with frequent facial palsy. The time between the onset of infectious and neurological symptoms suggests a postinfectious mechanism. Early diagnosis of GBS in COVID-19 patients is important as it might be associated with a severe disease course requiring intensive care and mechanical ventilation.

## RÉSUMÉ

Apparition du syndrome de Guillain-Barré à la suite d’une infection à la COVID-19 : une étude systématique.

**Contexte:**

C’est en janvier 2020 qu’on a documenté en Chine le premier cas de syndrome de Guillain-Barré (SGB) attribuable à une infection à la COVID-19. Le SGB est connu pour être post-infectieux et pour apparaître à la suite de plusieurs types d'infections. Bien qu’une réelle causalité puisse seulement être établie par l’entremise de vastes études épidémiologiques, nous nous sommes penchés sur cette association au moyen d’un examen approfondi de la littérature sur le sujet.

**Méthodes:**

Pour ce faire, nous avons interrogé les bases de données suivantes : PubMed, EMBASE et Google Scholar. À cet égard, nous avons inclus dans notre étude tous les articles complets rédigés en anglais ou en espagnol contenant des données originales à propos de patients atteints du SGB et ayant été infectés récemment à la COVID-19. Les variables qui nous ont le plus intéressés portaient sur leurs caractéristiques démographiques, sur les examens diagnostics qui avaient été effectués et sur la période de latence entre les symptômes dits « arboviraux » et ceux de nature neurologique. Davantage de variables ont été par la suite regroupées pour identifier les variantes cliniques et électro-physiologiques du SGB, les traitements utilisés et l’évolution de l’état de santé de ces patients. On a aussi pu valider la certitude d’un diagnostic de SGB à l’aide des critères de Brighton.

**Résultats:**

Au total, ce sont 109 cas de SGB que nous avons identifiés. De ce nombre, 99 étaient liés à des cas confirmés d’infection à la COVID-19, l’âge moyen des patients étant de 56,07 ans. La période moyenne de latence entre les premiers symptômes dits « arboviraux » et des manifestations neurologiques pour des cas confirmés d’infection à la COVID-19 a été de 12,2 jours. À noter que les variantes cliniques et électromyographiques prédominantes de la SGB ont relevé respectivement de la forme classique sensorimotrice et de la polyradiculonévrite inflammatoire démyélinisante associées à ce syndrome. Enfin, soulignons que 40 cas ont nécessité le recours aux soins intensifs, que 33 d’entre eux ont entraîné l’utilisation de la ventilation artificielle tandis que 6 autres se sont soldés par un décès.

**Conclusion:**

Il n’est pas rare que des études portant sur les liens entre le SGB et l’infection à la COVID-19 aient signalé un syndrome de type sensorimoteur démyélinisant avec de fréquentes manifestations de paralysie faciale. La période qui sépare une infection à la COVID-19 de l’apparition de symptômes neurologiques suggère ainsi un mécanisme post-infectieux. Un diagnostic précoce de SGB chez des patients infectés à la COVID-19 est donc important car un tel syndrome peut être associé à une évolution préoccupante de leur état de santé nécessitant des soins intensifs et une ventilation artificielle.

## Introduction

In December 2019, the COVID-19 epidemic emerged in Wuhan, China, causing global alterations not only in the field of healthcare, but also in all walks of life. The viral agent responsible for this clinical illness is described as severe acute respiratory syndrome coronavirus 2 (SARS-CoV-2). It was documented that SARS-CoV-2 is associated with neurologic manifestations, including headache, dizziness, hypogeusia, and hyposmia.^1^ Beside hypogeusia and hyposmia, there has been increased reporting of distinct peripheral nervous system (PNS) diseases in COVID-19 patients.

Guillain Barre syndrome (GBS) is an inflammatory disease of the PNS, characterized by rapidly progressive, symmetrical, and typically ascending weakness of the limbs with reduced or absent deep tendon reflexes, and upper and lower extremities non-length-dependent paresthesia and sensory symptoms at onset. Cranial nerves involvement can also be present in GBS patients, with facial and bulbar muscles often being affected.^2^ GBS can be classified into different distinct clinical variants including classical sensorimotor, paraparetic, pure motor, pure sensory, Miller Fisher syndrome (MFS), pharyngeal-cervical-brachial variant (PCB), bilateral facial palsy with paranesthesia, and Bickerstaff brainstem encephalitis.^3^ Another classification of GBS based on the electromyography (EMG) findings has also been described, with acute inflammatory demyelinating polyneuropathy (AIDP) being the most common variant. Other EMG variants of GBS according to this classification include acute motor axonal neuropathy (AMAN) and acute motor and sensory axonal neuropathy (AMSAN).^4^

GBS has been linked to a variety of causative pathogens; campylobacter jejuni (C. jejuni), cytomegalovirus (CMV), hepatitis E virus, mycoplasma pneumoniae, Epstein–Barr virus (EBV), and Zika virus.^5–8^ The emergence of Zika virus epidemic in 2016 was noticeably linked to increased incidence of GBS.^9^ GBS has also been linked to Middle East respiratory syndrome coronavirus (MERS-CoV) which is genetically similar to SARS-CoV-2 and was responsible for the outbreak of Middle East Respiratory Syndrome in 2013.^10^ In January 2020, the first case of GBS due to SARS-CoV-2 infection was documented in China.^11^ In this article, we are reviewing all the published cases of GBS that have been linked to SARS-CoV-2, to study their clinical presentations, the average latency period till the onset of GBS symptoms, the global distribution of these cases, and the findings of the ancillary GBS investigations.

## Methods

**Figure 1:**
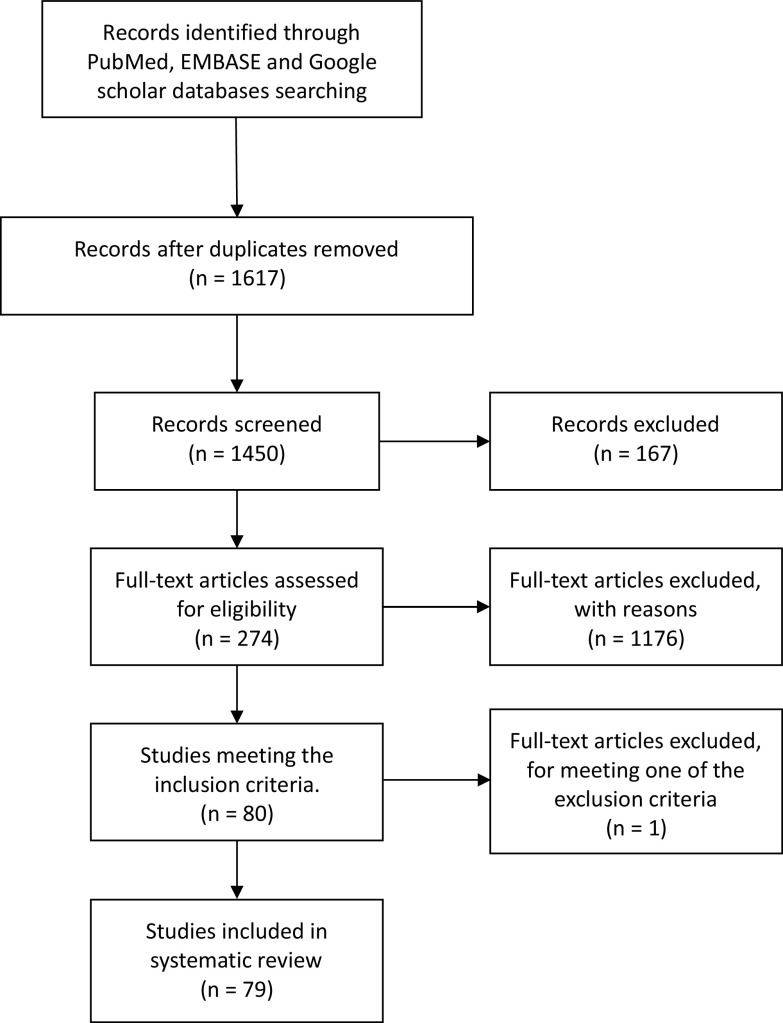
PRISMA figure showing the steps of literature search and paper selection for the systematic review.

We searched PubMed, EMBASE, and Google scholar and included all papers with full text available in English or Spanish and reporting original data of patients with GBS and recent COVID infection. This systematic literature review followed the Preferred Reporting Items for Systematic Reviews and Meta-Analyses (PRISMA) statement (Figure 1).^12^ We used the following keywords on our search: GBS, MFS, COVID-19, SARS-CoV2, and neurological manifestations, and these databases were searched from August 26, 2020 and to February 7, 2021. Titles and abstracts were screened by two researchers (M. Aladawi and M. Elfil). The full texts of the selected papers were read in full by five researchers (M. Aladawi, B. Abu-Esheh, D. Abu Jazar, A. Armouti, and A. Bayoumi), and their extracted data were then revised by M. Aladawi.

We included all papers, reports, or bulletins with the full text available in English or Spanish, reporting data of patients with GBS and a probable or confirmed recent COVID-19 diagnosis. Preidentified exclusion criteria were: (1) GBS with proven triggering infection other than SARS-CoV2 (e.g., C. jejuni), (2) presence of alternative diagnosis for weakness (e.g., critical illness neuropathy), and (3) latency period between COVID-19 infection and the onset of GBS symptoms of more than 6 weeks. Variables of interest were demographics, COVID-19 diagnostic investigations, latency between constitutional viral symptoms and neurological symptoms, presence of a negative SARS-Cov2 polymerase chain reaction (PCR) at the time of neurological manifestations (Table 1). Studied variables of cases with confirmed COVID-19 infection were pooled into another table to identify clinical characteristics (viral symptoms and neurological symptoms), GBS ancillary diagnostic investigations (cerebrospinal fluid [CSF] findings and testing for antiganglioside antibodies), the predominant clinical and electrophysiological variants of COVID-19-related GBS, received immunomodulatory therapy, disease progression, and clinical outcome (Table 2).

**Table 1:** Demographics, diagnostic confirmation of COVID-19, latency duration of neurologic symptoms, and PCR testing at the time of neurological manifestations of both suspected and confirmed cases of COVID-19

		COVID diagnostics at time of arboviral symptoms			Duration between arboviral and neurological symptoms	Negative repeat PCR at time of neurological symptoms	
Author	Country	PCR	Serology	Chest radiographic features		Nasopharyngeal swab	Cerebrospinal fluid
**Confirmed cases**							
Diez-Porras^14^	Spain	Yes	No	No	5 d	No	NA
Granger^15^	Italy	Yes	No	No	22 d	No	NA
Hirayama^16^	Japan	Yes	No	Yes	20 d	Yes	NA
Liberatore^17^	Italy	Yes	No	Yes	23 d	No	NA
Nanda^18^	India	Yes	No	No	10 d	No	NA
Nanda^18^	India	Yes	No	No	6 d	No	NA
Nanda^18^	India	Yes	No	No	7 d	No	NA
Nanda^18^	India	Yes	No	Yes	10 d	No	NA
Atakla^19^	Guinea	Yes	No	Yes	14 d	No	NA
Rajdev^20^	USA	Yes	No	Yes	18 d	No	NA
Senel^21^	Germany	Yes	Yes	No	NA	Yes	Yes
Tard^22^	France	Yes	No	Yes	10 d	No	NA
Chan^23^	USA	Yes	No	No	18 d	No	Yes
Chan^23^	USA	Yes	No	No	23 d	Yes	Yes
Sedaghat^24^	Iran	Yes	No	Yes	11 d	No	NA
Ebrahimzadeh^25^	Iran	Yes	No	Yes	18 d	No	NA
Ebrahimzadeh^25^	Iran	Yes	No	No	10 d	No	NA
Arnaud^26^	France	Yes	No	Yes	22 d	No	Yes
Paybast^27^	Iran	Yes	No	No	16 d	No	NA
Paybast^27^	Iran	Yes	No	No	19 d	No	NA
Coen^28^	Switzerland	Yes	Yes	No	6 d	No	Yes
Dinkin^29^	USA	Yes	No	No	4 d	No	NA
Dinkin^29^	USA	Yes	No	Yes	NA	No	NA
Manganotti^30^	Italy	Yes	No	No	18 d	No	Yes
Manganotti^30^	Italy	Yes	No	No	30 d	No	Yes
Manganotti^30^	Italy	Yes	No	No	14 d	No	Yes
Manganotti^30^	Italy	Yes	No	No	33 d	No	NA
Manganotti^30^	Italy	Yes	No	No	22 d	No	Yes
Fernández-Domínguez^31^	Spain	Yes	No	No	15 d	Yes	Yes
Hutchins^32^	USA	Yes	No	Yes	16 d	No	NA
Kilinc^33^	Netherlands	No	Yes	No	28 d	No	Yes
Naddaf^34^	USA	No	Yes	Yes	17 d	Yes	Yes
Abrams^35^	USA	Yes	No	Yes	10 d	No	Yes
Gigli^36^	Italy	No	Yes	Yes	17 d	Yes	NA
Bracaglia^37^	Italy	Yes	No	No	0 d	No	NA
Sidig^38^	Sudan	Yes	No	Yes	5 d	No	NA
Lascano^39^	Switzerland	Yes	Yes	No	15 d	No	Yes
Lascano^39^	Switzerland	Yes	No	No	7 d	No	NA
Lascano^39^	Switzerland	Yes	No	No	22 d	No	Yes
Camdessanche^40^	France	Yes	No	Yes	11 d	No	NA
Abolmaali^41^	Iran	Yes	No	Yes	0 d	No	NA
Abolmaali^41^	Iran	Yes	No	Yes	10 d	No	NA
Abolmaali^41^	Iran	Yes	No	Yes	9 d	No	NA
L.Chan^42^	Canada	Yes	No	Yes	0 d	No	Yes
Sancho-Saldaña^43^	Spain	Yes	No	No	15 d	No	Yes
Assini^44^	Italy	Yes	No	No	20 d	No	Yes
Assini^44^	Italy	Yes	No	Yes	23 d	No	Yes
Frank^45^	Brazil	Yes	Yes	No	5 d	No	Yes
Caamaño^46^	Spain	Yes	No	Yes	10 d	No	Yes
Oguz-Akarsu^47^	Turkey	Yes	No	Yes	0 d	No	Yes
Toscano^48^	Italy	Yes	No	Yes	7 d	No	Yes
Toscano^48^	Italy	Yes	No	No	10 d	No	Yes
Toscano^48^	Italy	Yes	No	Yes	10 d	No	Yes
Toscano^48^	Italy	Yes	No	No	5 d	No	Yes
Toscano^48^	Italy	No	Yes	Yes	7 d	Yes	Yes
Reyes-Bueno^49^	Spain	No	Yes	No	15 d	Yes	NA
Bigaut^50^	France	Yes	No	Yes	21 d	No	Yes
Bigaut^50^	France	Yes	No	Yes	10 d	No	Yes
Padroni^51^	Italy	Yes	No	No	24 d	Yes	NA
Tiet^52^	UK	Yes	No	No	14 d	No	Yes
Ameer^53^	UK	Yes	No	No	4 d before arboviral symptoms	No	Yes
Wada^54^	China	Yes	No	Yes	17 d	No	NA
Ray^55^	UK	Yes	No	No	0 d	No	NA
Guijarro-Castro^56^	Spain	Yes	No	Yes	21 d	No	NA
Gutiéerrez-Ortiz^57^	Spain	Yes	No	No	5 d	No	Yes
Gutiéerrez-Ortiz^57^	Spain	Yes	No	No	3 d	No	Yes
Agosti^58^	Italy	Yes	No	Yes	5 d	No	NA
Zhao^11^	China	Yes	No	Yes	8 d before arboviral symptoms	No	NA
Khalifa^59^	KSA	Yes	No	Yes	20 d	No	NA
Farzi^60^	Iran	Yes	No	Yes	10 d	No	NA
Alberti^61^	Italy	Yes	No	Yes	7 d	No	Yes
Sandeep^62^	US	Yes	No	Yes	14 d	Yes	NA
Korem^63^	USA	Yes	No	No	14 d	No	NA
Civardi^64^	Italy	Yes	No	No	10 d	No	Yes
Virani^65^	USA	Yes	No	No	10 d	No	NA
Khaja^66^	USA	Yes	No	No	0 d	No	Yes
Lampe^67^	Germany	Yes	No	No	2 d	No	NA
Ottaviani^68^	Italy	Yes	No	Yes	10 d	No	Yes
Scheidl^69^	Germany	Yes	No	No	3 weeks	Yes	NA
El Otmani^70^	France	Yes	No	Yes	13 d	No	Yes
Lantos^71^	USA	Yes	No	No	4 d	No	NA
Riva^72^	Italy	No	Yes	Yes	20 d	Yes	Yes
Helbok^73^	Austria	No	Yes	Yes	14 d	Yes	Yes
Webb^74^	UK	Yes	No	No	7 d	No	Yes
Pfefferkorn^75^	Germany	Yes	No	Yes	14 d	No	Yes
Dufour^76^	USA	Yes	No	No	21 d	Yes	NA
Jones^77^	UK	Yes	No	No	22 d	No	NA
Ghosh^78^	India	Yes	No	No	0 d	No	NA
Mackenzie^79^	Columbia	Yes	No	No	0 d	No	NA
Mansour^80^	Morroco	Yes	No	Yes	12 d	No	Yes
Petrelli^81^	Italy	Yes	No	No	15 d	No	NA
Yaqoob^82^	NA	Yes	No	Yes	12 d	No	NA
Bueso^83^	USA	Yes	No	Yes	22 d	No	NA
Manji^84^	Tanzania	Yes	No	No	7 d	No	NA
Su^85^	USA	Yes	No	Yes	6 d	No	Yes
Galán^86^	Spain	Yes	No	Yes	10 d	No	NA
Barrachina-Esteve^87^	Spain	Yes	No	Yes	0 d	No	Yes
Marta-Enguita^88^	Spain	Yes	No	Yes	8 d	No	NA
Gigli^89^	Italy	No	Yes	Yes	NA	Yes	Yes
**Suspected cases**							
Gigli^89^	Italy	No	No	Yes	NA	Yes	NA
Gigli^89^	Italy	No	No	No	NA	Yes	NA
Gigli^89^	Italy	No	No	No	NA	Yes	Yes
Gigli^89^	Italy	No	No	No	NA	Yes	Yes
Gigli^89^	Italy	No	No	No	NA	Yes	NA
Gigli^89^	Italy	No	No	No	NA	Yes	Yes
Gigli^89^	Italy	No	No	Yes	NA	Yes	NA
Manganotti^90^	Italy	No	No	No	16 d	No	NA
Gale^91^	UK	No	No	Yes	NA	Yes	NA
García-Manzanedo^92^	Spain	No	No	Yes	21 d	No	NA

**Table 2:** Demographics, clinical features, and GBS classification in patients with confirmed cases of COVID-19

Demographics	
Mean age (years)	56.07
Males	71
Females	28
Average latency of neurological symptoms (days)	12.2 (±7.5)
**Arboviral symptoms**	
Fever	67/95
Sore throat	12/95
Anosmia/dysgeusia	25/95
Dry cough	60/95
Rash	2/95
Arthralgia/myalgia	18/95
Chest pain	1/95
Shortness of breath	27/95
Headache	10/95
Gastrointestinal symptoms	17/95
**Neurological signs and symptoms**	
Dysphagia	18/99
Dysarthria	11/99
Sensory symptoms	65/99
Diplopia	11/99
Facial palsy	42/99
Bulbar palsy	12/99
Ocular palsy	11/99
Tetraparesis	64/99
Paraparesis	81/99
Sensory deficits	41/99
Areflexia or hyporeflexia	93/99
Ataxia	18/99
Respiratory dysfunction	30/99
Dysautonomia	20/99
**GBS clinical variant**	
Classical sensorimotor GBS	64/99
Paraparetic GBS	16/99
Miller Fisher syndrome	9/99
Pharyngeal-cervical-brachial GBS	2/99
Bilateral facial palsy with paranesthesia	3/99
Bickerstaff brainstem encephalitis	0/99
Pure motor GBS	0/99
Pure sensory GBS	1/99
Unclassified	4/99
**CSF analysis**	
Albuminocytologic dissociation	74/86
Oligoclonal bands	2/86
Normal	10/86
**Neuroimaging findings**	
Cranial nerve enhancement	9/61
Spinal nerve root enhancement	10/61
Unremarkable	44/61
**Antiganglioside antibodies**	
Anti-GM1	3/50
Anti-GM2	2/50
Anti-GD1a	3/50
Anti-GD1b	3/50
Anti-GD3	1/50
Anti-GQ1b	1/50
Anti-GT1b	1/50
Anti-Gal-C	1/50
Negative antiganglioside Ab	43/50
**GBS EMG variant**	
AIDP	59/77
AMAN	8/77
AMSAN	10/77
**Immunomodulatory treatment**	
IVIG	72/98
PLEX	10/98
IVIG and PLEX	7/98
No treatment	8/98
**Clinical outcome**	
ICU admission	40/99
Mechanical ventilation	33/99
Death	6/99
**Brighton criteria**	
Level 1–3	84/99
Level 4	9/99
Other variants	6/99

AIDP= acute inflammatory demyelinating polyneuropathy; AMAN=acute motor axonal neuropathy; AMSAN=acute motor and sensory axonal neuropathy; CSF=cerebrospinal fluid; GBS=Guillain Barre syndrome; ICU=intensive care unit; IVIG=intravenous immunoglobulin; PLEX=plasmapheresis.

Cases were classified according to the reported diagnostic certainty levels for GBS and COVID-19 infection. To classify the diagnosis of GBS, we employed the Brighton Collaboration Criteria.^13^ The diagnostic certainty of COVID-19 infection was classified as confirmed and suspected. As confirmed cases were identified by the presence of positive PCR at the time of arboviral symptoms or the presence of positive SARS-CoV2 antibodies whether during arboviral or neurological presentation as in some cases GBS was the presenting manifestation.

## Results

We identified 1450 articles in the databases researched, of which 79 papers were included in our systematic review (66 case reports and 13 cases series). The selected studies reported on a total of 109 GBS cases with a confirmed or a suspected COVID-19 infection. One case was excluded as it met one of the exclusion criteria; the latency between the onset of COVID-19 infection and the GBS onset of symptoms was 53 d (>6 weeks).^93^

The applied investigations in confirming COVID-19 infection at the time of arboviral symptoms were COVID-19 PCR testing, detection of SARS-CoV2 antibodies, and suggestive features on chest radiography. Cases with either positive PCR or SARS-CoV2 antibodies were categorized as confirmed cases, whereas patients diagnosed based on abnormal chest radiographs or clinical suspicion only were categorized as suspected cases. We have identified 99 cases of COVID-19 complicated by GBS that has been confirmed with either PCR testing or serology (Table 1). Table 1 also includes the latency period between arboviral symptoms and neurologic manifestations, the country of reported cases, and repeat COVID-19 PCR at the time of neurological symptoms either from nasopharyngeal, swabs, or in the CSF.

The global distribution of cases was as follows: 32 cases in Italy, 16 cases in the United States, 12 cases in Spain, 9 cases in Iran, 6 cases in France, 6 cases in the United Kingdom, 5 cases in India, 4 cases in Germany, 4 cases in Switzerland, 2 cases in China, 1 case in Guinea, 1 case in Austria, 1 case in Brazil, 1 case in Canada, 1 case in Columbia, 1 case in Japan, 1 case in Morocco, 1 case in Netherlands, 1 case in Sudan, 1 case in Tanzania, 1 case in Turkey, and 1 case in Saudi Arabia.

At the time of the patient’s demonstrated neurologic signs and symptoms, repeat SARS-CoV2 PCR swab was negative in 23 cases. Reverse transcription PCR (RT-PCR) for SARS-CoV-2 in the CSF was performed in 50 cases in which it was negative. The average latency period between the arboviral symptoms and neurologic manifestations for confirmed COVID-19 cases was 12.2 d (Table 2). There were two cases where neurological manifestations have preceded arboviral symptoms, and nine cases where patients only presented with neurologic deficits with no symptoms of COVID-19, but they had positive COVID-19 testing.

Table 2 shows the pooled data of GBS cases that have been preceded by a confirmed COVID-19 infection. There was a total of 99 cases (71 males and 28 females), the average age was 56.07 years. The most common arboviral symptoms prior to GBS were fever, dry cough, dyspnea, and gastrointestinal symptoms. There were four cases which did not report patient’s arboviral symptoms prior to GBS manifestations. The most commonly reported neurological signs and symptoms were ascending motor weakness (tetraparesis and paraparesis), diminished deep tendon reflexes, sensory disturbances (paresthesia), sensory loss, and facial palsy. GBS was complicated by respiratory failure in 30 cases and dysautonomia in 20 cases.

Clinical GBS variants have been identified in these cases. The most commonly reported GBS variants were classical sensorimotor GBS (64 cases), followed by paraparetic GBS (16 cases), MFS (9 cases), facial diplegia with paresthesia (3 cases), pharyngeal-cervical-brachial GBS (2 cases), and pure sensory GBS (1 case). There were four cases that could not be classified into any of the GBS clinical variants. CSF analysis was performed in 86 cases. Seventy-four cases have shown albuminocytologic dissociation (normal CSF protein <45 mg/dl^94^), 2 cases have shown oligoclonal band, and 10 cases had no abnormalities in the CSF analysis. Antiganglioside antibodies were investigated in 50 cases. The majority of cases had negative antiganglioside antibodies (43 cases). Each of anti-GM1, anti-GD1a, and anti-GD1b were positive in three cases; anti-GM2 was positive in two cases; and each of anti-GD3, anti-GQ1b, anti-GT1b, and anti-Gal-C were positive in one case.

Electromyography (EMG) was performed in 77 cases. The predominant EMG variant of GBS was AIDP (59 cases), followed by AMSAN (10 cases), and AMAN (8 cases). Eighty-nine reports confirmed the use of immunomodulatory treatment for GBS. Seventy-two cases received intravenous immunoglobulin (IVIG) therapy, 10 cases were treated with plasmapheresis (PLEX), and 7 cases were treated with both IVIG and PLEX. In terms of disease progression and the clinical outcomes, 40 cases required admission to the intensive care unit (ICU), 33 cases required mechanical ventilation, and 6 cases were complicated by death.

Brighton criteria were applied to improve the diagnostic certainty for the cases; valid symptomatology included bilateral and flaccid weakness of limbs at the time of presentation, decreased deep tendon reflexes in affected limbs, the presence of a monophasic course of neurologic symptoms, CSF cell count <50/μl, elevated CSF protein, EMG findings consistent with one of the subtypes of GBS, and the absence of alternative diagnosis. Accordingly, cases were classified from level 1–4 of diagnostic certainty.^13^ Cases with MFS where the complete triad of ophthalmoplegia, ataxia, and areflexia was not present were classified as level 4.^95^ Cases with other variants such as facial diplegia with paresthesia, PCB variant, and pure sensory GBS has been excluded. Accordingly, 51 cases have fulfilled level 1 of diagnostic certainty, 26 cases have fulfilled level 2, 7 cases have fulfilled level 3, and 9 cases fulfilled level 4. We have concluded that the reported cases have a high-diagnostic certainty of GBS as most of the cases have been classified into level 1–3 of Brighton criteria.

## Discussion

Our systematic review shows that the published literature on COVID-19-related GBS commonly report a classic sensorimotor variant of GBS with often facial palsy and a demyelinating electrophysiological subtype. The disease course is frequently severe with high rates of respiratory dysfunction and ICU admission.^96^ The time elapsed between infection and neurologic manifestations, and a negative PCR in spinal fluid might suggest that there is a postinfectious mechanism implicated in the etiology of COVID-19-related GBS. However, these results should be interpreted with caution as the cases included in this systematic review varied widely in diagnostic ascertainment and reporting of different variables. Moreover, the reported cases were limited to certain geographical areas, which might provide a source of bias.

The constellation of sensorimotor signs with facial palsy, respiratory insufficiency, and a demyelinating electrophysiological subtype has been described in GBS patients with other viral infections such as CMV and Zika virus, which might indicate that this clinical and electrophysiological variant of GBS is related to viral infections in general.^8,97^ On the other hand, C. jejuni is typically associated with pure motor and axonal type of GBS.^98^ Although GBS is generally more common in men as compared with women,^99^ in our systematic review, we have found that the male to female ratio was 2.5:1 which is significantly higher than what is usually reported.^100^ This suggests that men might be more prone to COVID-19-related GBS.

In our review, the most common arboviral symptoms were fever and dry cough, which is typical in COVID-19 infection.^101^ We could not identify a specific arboviral symptom that could be typically preceding the development of GBS. However, we have identified two cases in which GBS manifestations preceded COVID-19 arboviral symptoms, and nine cases that did not present with arboviral symptoms initially. This chronology of GBS preceding the arboviral symptoms has not been previously reported with GBS related to other viral agents. In addition, the asymptomatic infection of COVID-19 might limit the ability to accurately determine the latency period between viral symptoms and the GBS presentation.

The mean duration between the onset of COVID-19 infectious symptoms and GBS presentation was 2 weeks, which is similar to other infections preceding GBS.^102^ The latency between COVID-19 infection and GBS was more than a week for most cases, but it should be taken into consideration that COVID-19 can initially be asymptomatic which makes the latency duration arguably longer than reported. This suggests a postinfectious immunopathogenesis rather than direct neuronal damage or a parainfectious mechanism. The fact that COVID-19 PCR of the CSF was not positive in a single report, the negativity of repeat nasopharyngeal PCR at the time of symptoms in almost one-third of the cases, and the absence of elevated white blood cell count in the CSF in majority of cases, further argues against the assumption of COVID-19 infection being directly responsible for the GBS development in this proportion of patients.

Despite the fact that previous epidemiological studies have suggested that COVID-19 might not be associated with GBS,^103^ the chronology of publication of the COVID-19-related GBS cases followed the same pattern of the global spread of COVID-19, as the first cases report was from China followed by Italy, Iran, and USA indicates a positive association.^11,24,48,65^ GBS has been historically related to various pathogens including C. jejuni, M. pneumoniae, EBV, CMV, Hepatitis E virus, and Zika virus.^5–9^ However, in certain pathogens such as Hepatitis E virus, this association has not been established globally, as it was only reported in Netherlands and Bangladesh.^104^ Therefore, immunogenicity of COVID-19 in the development of GBS should consider the variations between different populations,^105–108^ as epidemiologic studies involving certain populations might introduce bias in reporting results.

Interestingly, almost half of the cases were tested for the presence of antiganglioside antibodies in serum. There were only seven cases have tested positive for different antiganglioside antibodies. Historically, different antigangliosides have been linked to different variants of GBS, such as anti-GQ1b in MFS and anti-GD1a in PCB variant.^109,110^ Antiganglioside antibodies are considered to be biomarkers of axonal injury rather demyelination, as they directly target the neuronal membrane gangliosides.^111^ Because most of the COVID-19-related GBS cases reported a demyelinating variant of GBS, it can be anticipated that the presence of antiganglioside antibodies would be low. Thus, the spectrum of immune cascade in COVID-19-related GBS should be expanded by studying other different antibodies affecting the myelin sheath, Schwann cell components, and the neuronal axolemma.^112,113^ One case was reported with positive NF-155 and NF-186 antibodies, which are structural proteins in the node of Ranvier.^22^

The possible role of host immunogenetic background in the development of GBS and its variants has been related to human leukocyte antigen (HLA) polymorphism in different populations, this observation might explain the increased reporting of COVID-19 related GBS in the Italy, as one-third of the cases identified in our review were Italian.^114,115^ The role of HLA polymorphism in COVID-19 related GBS has been emphasized in one of the cases reported by Gigli et al.,^36^ in which SARS-CoV2 antibodies were detected in the CSF. Interestingly, HLA analysis of the reported case showed several HLA alleles that are known to be associated with GBS, such as: HLA-A33,^116^ DRB1 * 03:01,^117^ and DQB1 * 05:01.^118^

With the emergence of COVID-19 pandemic, there have been increasing reports of various neurological complications in infected patients, which was well documented and studied in other coronaviruses.^1^ Genomic analysis shows that SARS-CoV-2 is in the same beta-coronavirus (βCoV) clade as MERS-CoV and SARS-CoV, and shares a highly homological sequence with SARS-CoV.^119^ There has been clinical evidence of neuromuscular sequela in SARS CoV and MERS infection and the most documented neuromuscular syndromes related to these viruses are critical illness polyneuropathy and myopathy, which are hypothesized to occur in the context of severe inflammatory response syndrome (SIRS).^120^ Cases of MERS-related GBS have been reported, yet GBS in these cases has been linked to the treatment received for MERS infection, such as interferon alpha2 and Lopinavir/ritonavir.^10^ In contrast to MERS, SARS-CoV2 is likely associated with GBS.

## Conclusion

Based on this systematic review, most cases of COVID-19-related GBS are of the sensorimotor demyelinating subtype with frequent facial palsy. The latency between infection and onset of neurologic symptoms as well as the absence of viral genome detected by PCR suggest a postinfectious, rather than a direct infectious or para-infectious mechanism. Global reporting of COVID-19-related GBS cases, in addition to testing for different antibodies to different structural proteins and glycolipids in the peripheral nerves, would improve the understanding of the immunological cascade of COVID-19-related GBS. Finally, early diagnosis and identification of GBS in COVID-19 patients is important as COVID-19-related GBS might be associated with a severe disease course that frequently requires ICU admission and mechanical ventilation.
